# Self-Mutilating Lesions Associated with Rabies in White-Tailed Deer (*Odocoileus virginianus*): Detection and Diagnosis

**DOI:** 10.3390/pathogens15070715

**Published:** 2026-07-07

**Authors:** Madison R. Stevens, Kristin Hamaker, Ian Gereg, Matthew Shaub, Lane Potts, Erica A. Miller, Taylor C. Chan, Madeline Vile, Andrew Di Salvo, Kevin D. Niedringhaus

**Affiliations:** 1 Wildlife Futures Program, School of Veterinary Medicine, University of Pennsylvania, Kennett Square, PA 19348, USA; 2 Pennsylvania Veterinary Laboratory, Pennsylvania Department of Agriculture, Harrisburg, PA 17110, USA; 3 Pennsylvania Game Commission, Harrisburg, PA 17110, USA

**Keywords:** deer, encephalitis, rabies, wildlife health, zoonosis

## Abstract

Rabies is a universally fatal viral disease that affects a wide variety of mammalian species, including carnivores and herbivores. While testing and confirmed reports in wild carnivores are common, comparatively less is known about this disease in wild herbivores. Between 2022 and 2024, nine white-tailed deer (*Odocoileus virginianus*) in Pennsylvania, USA were diagnosed with rabies. Eight (89%) presented with erratic neurologic signs and one was found dead. Evidence of chronic self-mutilation characterized by hair loss and thickened, hardened, and hyperpigmented skin was present on the head in seven cases (78%) and/or body or legs in two cases (22%), while two (22%) did not have any evidence of external lesions. Microscopically, lesions were like those in other mammalian species, with variably severe inflammation and abundant cytoplasmic inclusion bodies with associated antigen immunoreactivity. This case series highlights a common and characteristic gross lesion that can prompt caution and testing for rabies in deer and further expands understanding of this disease in an uncommonly diagnosed species.

## 1. Introduction

Rabies is a global, fatal disease of mammals caused by a viral infection that is predominantly transmitted via the bite of an infected animal [1]. The incubation period between infection and the development of neurologic signs and death can vary widely but is most often between 30 and 90 days in most species [2,3]. In the United States, the most common wildlife taxa known to harbor and transmit rabies virus are bats, raccoons, skunks, foxes, and coyotes, but other carnivorous and herbivorous mammals have been documented with the disease [4]. Common rabies-vector species that display neurologic signs, particularly aggression, are often reported and submitted for rabies testing. However, recognition of rabies infections in herbivorous animals, including deer, is more challenging and often overlooked due to the historically less common reports of rabies in these species, a higher likelihood of other well-known important and often reportable infections, and less familiarity with characteristic clinical signs when aggression is not observed. Recognizing the risk of rabies in herbivores in general, and deer specifically, would benefit those handling these animals to reduce the risk of human exposure to this zoonotic virus.

## 2. Materials and Methods

From October 2022 through July 2024, 310 suspected diseased free-ranging white-tailed deer (*Odocoileus virginianus*) were encountered in the wild, humanely dispatched by gunshot if not already deceased, and submitted to the pathology service at the University of Pennsylvania Wildlife Futures Program at the Pennsylvania Animal Diagnostic Laboratory System (PADLS)-New Bolton Center for postmortem diagnostics. On gross examination, the head, when available, was assessed for any signs of trauma, infection, or other lesions. In cases of known neurologic signs (e.g., circling, head tilt, lack of fear) or unknown causes of morbidity or mortality, the head was split along the sagittal plane to examine and remove the brain using a hand saw to avoid aerosolizing any infectious pathogens and to preserve and subsequently isolate the caudal half of the cerebellum and brainstem. Unless gross examination revealed a clear cause of morbidity or mortality (e.g., brain abscess, acute trauma with hemorrhage), rabies testing was pursued (205 cases). Age was established via dentition and organized into recognized age classes of fawn (≈6 months), yearling (≈18 months), and adult (≈30+ months). Chronic wasting disease testing was also performed in all cases per established state surveillance guidelines.

In all cases, the sample for rabies testing was the caudal half of the cerebellum and associated brainstem, which was submitted to another PADLS branch, the Pennsylvania Veterinary Laboratory (PVL), an American Association of Veterinary Laboratory Diagnosticians (AAVLD)-accredited laboratory. Testing for rabies followed Centers for Disease Control laboratory methods utilizing the direct fluorescent antibody test (DFAT). Following the rabies specimen extraction, the remainder of the brain was placed into 10% neutral buffered formalin for subsequent histological preparations. Tissue was trimmed, embedded in paraffin wax, cut into 5 µm thick sections, mounted on glass slides and stained with hematoxylin and eosin. Unstained slides of brain tissue from two rabies cases were submitted to the Athens Veterinary Diagnostic Laboratory for rabies immunohistochemistry using AAVLD-established and approved protocols. All slides were reviewed by light microscopy by a board-certified veterinary pathologist.

## 3. Results

Between October 2022 and July 2024, nine deer (3.0% of all deer case submissions during this timeframe) were diagnosed with rabies in Pennsylvania. The nine cases out of 205 rabies tests performed resulted in 4.3% positive rabies results during the study period. Table 1 summarizes the history, location, demographic information, and any external gross lesions observed in these animals. Rabies-positive diagnoses were distributed throughout the years as follows: 2022 (n = 2), 2023 (n = 4), 2024 (n = 3). Seven out of the nine cases (78%) were adults, and the remaining two cases (22%) were yearlings. Seven cases (78%) were female and two (22%) were male. Submissions originated from seven unique counties in Pennsylvania, but two cases from 2022 were submitted within two days from the same county (Dauphin).

In seven of the nine cases (78%), there were varying severity and chronicity of abrasions on the skin characterized by hair loss and thickened and pigmented epidermis resulting in a ‘leathery’ appearance. These lesions were limited to just the head in five cases (56%) and head and legs in two cases (22%). Abrasions on the head were primarily localized to the dorsal calvarium when intact antlers in bucks limited the physical surface area (Figure 1A) and were much more widespread on the dorsal and lateral head in antlerless males and does (Figure 1B). The eyelids were markedly swollen, soft, and occluded the cornea with associated alopecia of the eyelids (Figure 1C) in three cases (33%). The leg of one deer had severe skin, fascia, and muscle loss resulting in exposed bone with marked alopecia remaining on adjacent intact skin (Figure 1D). Considering the lesions were most often localized to the dorsal head and lacked crusting, serous or suppurative exudate, or other evidence of active infection, and occasionally extended deep into the muscle, a presumptive cause of chronic surface abrasions was made. The lesions are interpreted to be self-induced due to known historical pruritic manifestations of viral and prion encephalitides, including the rabies virus, in ruminants and other animals [5,6,7].

Eight of the nine cases (89%) had abnormal behavior or neurologic signs reported or observed prior to mortality, and one (11%) deer was found dead with no antemortem behavioral signs reported. Four of the eight cases (50%) with documented antemortem clinical signs as summarized included erratic behavior such as pedaling on the ground and running into trees and/or buildings (2; 22%). Two cases (22%) had more subdued antemortem behavior with reports of bedding or lying down but being unresponsive to stimulation by humans; these latter two cases lacked the characteristic chronic skin wounds described above with skin being grossly unremarkable. None of the histories of rabies-positive deer described increased aggression. Co-morbidities were detected in two cases, one deer had meningitis associated with *Parelaphostrongylus tenuis* infection histologically and another was positive for the chronic wasting disease prion, as confirmed by enzyme-linked immunosorbent assay and immunohistochemistry.

Microscopic examination of the six brains highlighted a range of severity of inflammation with two (33%) having a paucity of perivascular cuffs and gliosis while the remaining four (67%) had abundant lymphoplasmacytic inflammation (Figure 2A). In all six cases where histopathology was performed, eosinophilic intraneuronal inclusion bodies (i.e., Negri bodies) were abundant and easily recognized within the hypothalamus, hippocampus (Figure 2B), cerebellar Purkinje cells (Figure 2C), and brainstem. The two cases with rabies immunohistochemistry performed on the brain showed widespread immunoreactivity including punctate staining in neuronal cytoplasm (Figure 2D), most common and severe in the brainstem and hippocampus but also in the cerebrum and cerebellum.

## 4. Discussion

This case series expands on another case series describing similar gross lesions and clinical signs in nine white-tailed deer with rabies in multiple North American states between 2001 and 2021 (Georgia, Maryland, North Carolina, Pennsylvania, and West Virginia) [8]. When combining those cases with ours, chronic and presumed self-induced abrasions on the head and around the eyes and/or legs appear common in white-tailed deer with rabies virus infections. Since our study, dissimilar to Weyna et al. 2022 [8], included all deer diagnosed with rabies regardless of presentation, we emphasize that this potentially characteristic gross lesion in rabid deer is common in those infected, but deer can still be positive for rabies virus without these external lesions. The two cases in our study that did not have the extent of severe, chronic dermal abrasions on the head or legs were also ones with more subtle neurologic signs and were not seen paddling or running into objects; rather, they were bedded down and did not respond to human presence. These signs may reflect a more recent infection early in the progression of the disease [9] or may reflect true differences in how individual deer respond to viral infection.

We have interpreted these gross lesions as self-inflicted due to their locations on the body and the behavior observed in the animals with clinical history; however, it is important to state that only one white-tailed deer (case number 6, Table 1) was directly observed to be displaying the repetitive traumatic behavior that resulted in the gross lesions on its legs and head. Nonetheless, the gross lesions observed in the remaining animals are consistent with prolonged head rubbing or butting more than other primary skin infections.

In 2022 and 2023, when national rabies data were available, North America saw an increase in the number of animals submitted for rabies testing and the number of detections [10,11]. In 2022, there were five deer reported as rabies-positive, in Oklahoma (n = 1), New York (n = 3), and Pennsylvania (n = 2) [10]. In 2023 there were 11 deer reported as rabies-positive, in Florida (n = 1), Georgia (n = 1), North Carolina (n = 2), New York (n = 3), and Pennsylvania (n = 4) [11]. The highest number of rabies detections in deer was Pennsylvania and New York in both years, which may suggest that even though deer can and do contract rabies, it may be more common in this species in the northeastern part of the country where rabies generally is more common in all wildlife species [10,11].

Potential reporting bias occurs as members of the public must contact the state wildlife agency dispatch service to report a diseased or deceased wild animal; therefore, there is generally an association between areas of greater human population and/or urban-wildlife interface and number of case submissions. Seasonality can also affect viable case submission. Postmortem condition can influence the ability and feasibility of successful ancillary diagnostics, and summer months can be more challenging to preserve sample quality due to ambient temperature and greater ectoparasitic activity. Cases with suspected or confirmed domestic animal or human exposure are prioritized for rabies testing and often are sent directly to the diagnostic laboratories overseen by the Pennsylvania Departments of Agriculture and Health, while animals with no human or domestic animal exposure, unknown or vague clinical history, or animals found dead are more likely to be submitted to our service. Some cases are submitted directly to the Pennsylvania Veterinary Laboratory by nuisance wildlife professionals, veterinarians, and the public without gross dissection and evaluation by our service. This accounts for potential discrepancies between our rabies detection information in deer and published national records. While our pathology and field team have worked to record instances of these gross presentations and subsequent detection of rabies in deer, it is possible that some cases go unreported or are submitted to other laboratories where we cannot evaluate the presence or absence of these potentially characteristic lesions.

In multiple rabies vaccine trial protocols, the furious form of rabies manifested in 43% of infected horses [12], 70% of cattle, and 80% of sheep [13], but domestic livestock are known to develop both the ‘furious’ and ‘dumb’ form of the disease [5]. A 1992 study with 10 sheep only had two individuals showing clinical signs of “emaciation, nervous reactions, and prostration before death” [14]. While overt aggression was not described as an antemortem behavior in the deer from our study, deer, like cattle and other herbivores, could still display aggressive behavior even as a spillover or dead-end host [9]. Anecdotally, deer with history of aggressive behavior on this service are typically habituated to humans and do not reflect a true infection or pathological central nervous system lesions.

Histological evaluation of the brain with or without immunohistochemistry was also shown to be a useful diagnostic tool if the rabies DFAT cannot be performed or when rabies is only recognized or suspected after the brain has been fixed in formalin. While the extent of inflammation was variable, the presence of intracytoplasmic inclusion bodies in neurons was consistent and common across all cases, making this a potentially reliable lesion to correlate with other testing modalities. Immunoreactivity was strong and consistent in all examined parts of the brain, including both the brainstem and cerebellum, as well as the hippocampus. Rabies immunoreactivity in the cerebellum and hippocampus expands the limited knowledge of the neuroanatomical location of rabies virus infection in white-tailed deer beyond a single known study [15]; investigating neuroanatomical localization with immunohistochemistry would be beneficial in future cases.

While rabies is predominantly detected in bats and carnivorous mammals in North America, this case series demonstrates detections in an atypical wildlife species that is abundant across the landscape. Rabies typing was not performed on these cases. Weyna et al. (2022) [8] highlighted that the typing of all rabid deer in a similar study was consistent with the carnivorous reservoir in their particular region, notably raccoons in eastern North America. The raccoon variant predominates among nonvalent rabies in Pennsylvania and is presumably the type associated with infection in the deer in this report but remains unconfirmed in these cases. While the risk of spillover from deer to other animals is unknown, any humans handling live or dead deer should be aware of these gross lesions that may serve as a useful clinical indicator to take precautions to reduce their exposure risk.

## Figures and Tables

**Figure 1:**
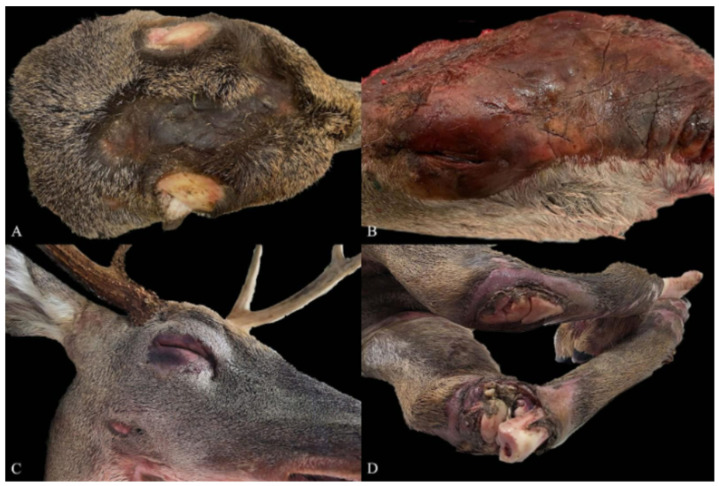
Gross lesions in three white-tailed deer showing chronic abrasions on the top of the skull (**A**,**B**), over the eyes (**B**,**C**) and on the legs with exposed bone (**D**).

**Figure 2:**
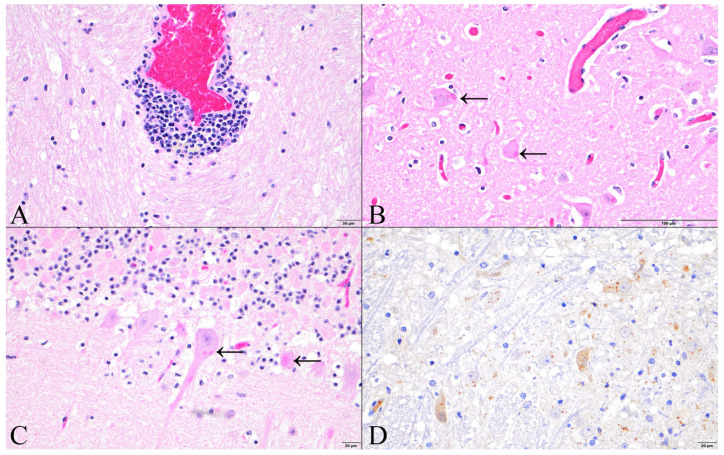
Microscopic lesions in the brains of rabid white-tailed deer. Lymphoplasmacytic perivascular cuffs were either rare or common and pronounced (**A**) while intra-neuronal inclusion bodies (Negri bodies; arrows) were common in all cases, most often in the hippocampus (**B**) and cerebellum (**C**). The two cases with rabies immunohistochemistry showed punctate intra-cytoplasmic immunoreactivity within the cytoplasm of neurons in the cerebrum (**D**), hippocampus, cerebellum, and brainstem.

**Table 1:** Summary of findings in white-tailed deer submitted to the Wildlife Futures Program, University of Pennsylvania School of Veterinary Medicine and diagnosed with rabies from 2022 to 2024. All animals were dispatched by PGC state game wardens except for case number 2 that was found dead. The clinical history is reported by initial reporting party and is often incomplete regarding all lesions involved or later documented at the laboratory.

Case Number	Date of Submission Day Month Year	Age	Sex	County	Clinical History
1	7 October 2022	Adult	F	Dauphin	Pedaling legs, unresponsive, discolored eyes ^1^.
2	10 October 2022	Adult	M	Dauphin	Found dead, worn skin on head and legs.
3	21 March 2023	Yearling	F	Bedford	Running in circles into trees and buildings, head swollen and hairless, eyes swollen shut ^2^.
4	30 June 2023	Adult	F	Jefferson	Bedded, unable to move, unresponsive, yellow ocular and nasal discharge.
5	20 September 2023	Adult	F	Jefferson	Bedded, unresponsive, worn dentition.
6	15 September 2023	Adult	F	Delaware	Severe self-mutilation, repetitive rubbing of limbs and head, later unresponsive, bedded in stream, and deceased.
7	29 April 2024	Adult	F	Perry	Struggling to walk, then bedded in creek, head and neck swollen and hairless, deceased upon arrival.
8	23 June 2024	Yearling	F	Bradford	Unafraid of humans, unresponsive, excess salivation.
9	9 July 2024	Adult	F	Juniata	Walking in circles, partially submerged in water, alopecia and discoloration present on head and neck, eyes swollen shut, responsive but unable to see.

^1^ *Parelaphostrongylus tenuis* associated inflammation. ^2^ Chronic Wasting Disease prion detected.

## Data Availability

Data supporting reported results are available from the corresponding author upon reasonable request.

## Abbreviations

The following abbreviations are used in this manuscript:

AAVLD	American Association of Veterinary Laboratory Diagnosticians
CDC	Centers for Disease Control and Prevention
PADLS	Pennsylvania Animal Diagnostic Laboratory System
CWD	Chronic wasting disease
